# Mechanistic link between diesel exhaust particles and respiratory reflexes

**DOI:** 10.1016/j.jaci.2017.04.038

**Published:** 2018-03

**Authors:** Ryan K. Robinson, Mark A. Birrell, John J. Adcock, Michael A. Wortley, Eric D. Dubuis, Shu Chen, Catriona M. McGilvery, Sheng Hu, Milo S.P. Shaffer, Sara J. Bonvini, Sarah A. Maher, Ian S. Mudway, Alexandra E. Porter, Chris Carlsten, Teresa D. Tetley, Maria G. Belvisi

**Affiliations:** a Respiratory Pharmacology Group, Airway Disease, National Heart & Lung Institute, Imperial College London, London, United Kingdom; c Department of Materials and London Centre for Nanotechnology, Imperial College London, London, United Kingdom; d Department of Chemistry and London Centre for Nanotechnology, Imperial College London, London, United Kingdom; h Lung Cell Biology, Airways Disease, National Heart & Lung Institute, Imperial College London, London, United Kingdom; b MRC & Asthma UK Centre in Allergic Mechanisms of Asthma, London, United Kingdom; e MRC-PHE Centre for Environment and Health, King's College London, London, United Kingdom; f NIHR Health Protection Research Unit in Health Impact of Environmental Hazards, London, United Kingdom; g Centre for Heart Lung Innovation, University of British Columbia, Vancouver, British Columbia, Canada

**Keywords:** Pollution, oxidative stress, transient receptor potential ion channels, sensory nerves, vagus, AhR, Aryl hydrocarbon receptor, DCM, Dichloromethane, DEP, Diesel exhaust particle, DLS, Dynamic light scattering, DMSO, Dimethyl sulfoxide, EDX, Energy-dispersive x-ray spectroscopy, IIAM, International Institute for the Advancement of Medicine, MitoTEMPO, (2-[2,2,6,6-Tetramethylpiperidin-1-oxyl-4-ylamino]-2-oxoethyl)triphenylphosphonium chloride, NAC, N-acetyl cysteine, NIST, National Institute of Standards and Technology, PAH, Polycyclic aromatic hydrocarbon, PM, Particulate matter, P_T_, Tracheal pressure, RAR, Rapidly adapting receptor, ROS, Reactive oxygen species, TEM, Transmission electron microscopy, TGA, Thermogravimetric analysis, TRP, Transient receptor potential, TRPA1, Transient receptor potential ankyrin-1, TTX, Tetrodotoxin, WT, Wild-type

## Abstract

**Background:**

Diesel exhaust particles (DEPs) are a major component of particulate matter in Europe's largest cities, and epidemiologic evidence links exposure with respiratory symptoms and asthma exacerbations. Respiratory reflexes are responsible for symptoms and are regulated by vagal afferent nerves, which innervate the airway. It is not known how DEP exposure activates airway afferents to elicit symptoms, such as cough and bronchospasm.

**Objective:**

We sought to identify the mechanisms involved in activation of airway sensory afferents by DEPs.

**Methods:**

In this study we use *in vitro* and *in vivo* electrophysiologic techniques, including a unique model that assesses depolarization (a marker of sensory nerve activation) of human vagus.

**Results:**

We demonstrate a direct interaction between DEP and airway C-fiber afferents. In anesthetized guinea pigs intratracheal administration of DEPs activated airway C-fibers. The organic extract (DEP-OE) and not the cleaned particles evoked depolarization of guinea pig and human vagus, and this was inhibited by a transient receptor potential ankyrin-1 antagonist and the antioxidant N-acetyl cysteine. Polycyclic aromatic hydrocarbons, major constituents of DEPs, were implicated in this process through activation of the aryl hydrocarbon receptor and subsequent mitochondrial reactive oxygen species production, which is known to activate transient receptor potential ankyrin-1 on nociceptive C-fibers.

**Conclusions:**

This study provides the first mechanistic insights into how exposure to urban air pollution leads to activation of guinea pig and human sensory nerves, which are responsible for respiratory symptoms. Mechanistic information will enable the development of appropriate therapeutic interventions and mitigation strategies for those susceptible subjects who are most at risk.

## Graphical abstract

**Figure:**
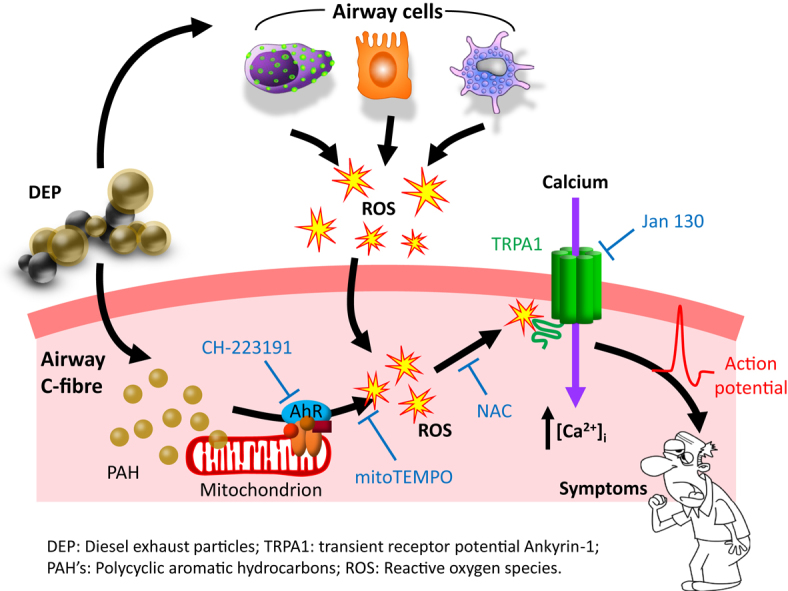

Air pollution is a major global health concern, especially in industrialized countries.^1^ In urban environments exposure to traffic-derived particulate matter (PM) has been a major focus, especially with regard to primary tailpipe emissions from diesel vehicles. Because of size and low density, smaller PM fractions are able to remain airborne, disperse widely in the environment, and penetrate deep into the lungs when inhaled to distribute throughout the respiratory tract. There is currently no safe lower limit of exposure to PM. Diesel exhaust particles (DEPs) represent a significant proportion of urban PM,^2^, ^3^ especially within Europe, because of the high proportion of diesel vehicles^4^ and ongoing problems with emission compliance.^5^ Epidemiologic studies have found strong associations between exposure to DEPs or air pollution markers indicative of diesel exhaust (black and elemental carbon) and respiratory symptoms, including cough, wheeze, and shortness of breath^6^, ^7^; hospital admissions^8^; and mortality.^9^ Clinical studies using diesel exposure have documented increases in total symptom scores^10^, ^11^ and increased airway resistance.^12^ However, information regarding the molecular mechanism linking DEP exposure and respiratory symptoms is lacking.

Respiratory reflexes are responsible for symptoms and regulated by vagal afferent nerves, which innervate the airway.^13^, ^14^, ^15^ There are several different sensory nerve subtypes present in the lung; some are more mechanically sensitive, and others are more chemosensitive, namely C-fibers and Aδ-fibers, respectively.

Transient receptor potential (TRP) channels present on vagus nerve termini situated in and under the airway epithelium can be activated by a wide variety of stimuli to elicit reflexes, leading to respiratory symptoms. These include mechanical and inflammatory stimuli, environmental irritants, and changes in osmolarity, pH, or temperature.^16^, ^17^ On activation, TRP channels allow influx of calcium into the cell, leading to subsequent membrane depolarization and ultimately generation of an action potential that propagates along the vagus nerve.^18^ Interestingly, one publication has demonstrated DEP-induced activation of TRPV4 expressed in an epithelial cell line, and another showed activation of TRPA1 on murine dorsal root ganglion cells.^19^, ^20^

Our hypothesis was that DEPs are able to initiate respiratory symptoms through direct activation of lung-specific afferent sensory nerves. The scope of this study was to determine whether DEPs can directly activate airway sensory nerves by using a range of human and animal *in vitro* models and *in vivo* electrophysiologic studies in an animal model. We also evaluated which component of DEPs was responsible and the signaling mechanisms involved.

## Methods

Detailed methods are provided in the Methods section in this article's Online Repository at www.jacionline.org.

### Animals

Male Dunkin-Hartley guinea pigs and C57BL/6 mice were used. All experiments were performed in accordance with the U.K. Home Office guidelines for animal welfare based on the Animals (Scientific Procedures) Act of 1986 and the Animal Research: Reporting of *In Vivo* Experiments guidelines.^21^

### Human tissue and ethics

Human lungs and tracheas surplus to transplantation requirements (n = 3, 56-73 years old; 1 male/2 female; 1 smoker/2 nonsmokers) with the vagus nerve still attached were used to obtain translational data to complement data generated in guinea pig tissue. Tissue was provided by the International Institute for the Advancement of Medicine (IIAM, Edison, NJ). In all cases the tissue was approved for use in scientific research, and ethical approval was obtained from the Royal Brompton & Harefield Trust.

### Compounds and materials

DEPs from a forklift truck (DEP SRM-2975) and its commercial organic extract (DEP-OE SRM-1975) were purchased from the National Institute of Standards and Technology (NIST, Gaithersburg, Md). Generator DEPs, obtained from the Air Pollution Exposure Laboratory, was obtained, and these have been designed for controlled inhalation of human subjects to aged and diluted diesel exhaust to mimic real-world occupational and environmental conditions.^22^ Drugs (listed in the Methods section in this article's Online Repository) were made up in stock solutions by using dimethyl sulfoxide (DMSO), with the final concentration of DMSO kept at 0.1% for experiments.

### Particle suspensions

Particle suspension solutions were prepared freshly daily. Suspensions of DEPs or cleaned particulate carbon core (par-DEPs) were prepared in modified Krebs-Henseleit solution by means of sonication before dilution to working concentrations. For *in vivo* experiments, suspensions were prepared in PBS in a similar manner.

### Physicochemical characterization of DEPs

Cryopreparation was done with an automatic plunge freezer. Nanoparticles dispersed in 1 μg/mL in Krebs-Henseleit solution were dropped onto a grid and frozen by rapidly plunging them into liquid ethane. These were transferred in their frozen state into a cryo-rod and then into the electron microscope. For chemical analysis, DEP samples were dispersed by means of sonication in ethanol and then pipetted onto a grid at room temperature. Transmission electron microscopy (TEM) and energy-dispersive x-ray spectroscopy (EDX) analyses were performed. The organic/inorganic ratio composition of SRM 2975 was assessed by using thermogravimetric analysis (TGA). Dynamic light scattering (DLS) measurements were also carried out, as described in the Methods section in this article's Online Repository. DEPs were separated into the organic extract (org-DEP) and cleaned particles (par-DEP) by using Soxhlet extraction.

### *In vivo* recording of action potential firing in single-fiber afferents

Guinea pigs were anesthetized with urethane (1.5 g/kg) intraperitoneally. The trachea was cannulated, and the animal was artificially ventilated. The right jugular vein and carotid artery were cannulated for injecting drugs and measuring systemic arterial blood pressure, respectively. Animals were paralyzed with vecuronium bromide that was initially administered intravenously at a dose of 0.10 mg/kg, followed every 20 minutes by 0.05 mg/kg administered intravenously to maintain paralysis. The depth of anesthesia was frequently assessed by monitoring the response of heart rate and blood pressure to noxious stimuli (as described below). Both cervical vagus nerves were located through a cervical incision, dissected free, and cut at the central end. The left vagus nerve was used for sensory nerve fiber recording, as previously described (diagram of experimental setup can be found in a recent review article^16^).^23^ After identification of a suitable single nerve fiber, control responses were obtained to capsaicin (100 μmol/L in saline, aerosolized for 15 seconds), acrolein (10 mmol/L in saline, aerosolized for 60 seconds), and citric acid (300 mmol/L, aerosolized for 60 seconds). The nerve under investigation was then challenged with either vehicle (PBS, 200 μL) or DEPs (10 μg/mL in PBS, 200 μL, intratracheal dose), and subsequent action potentials were recorded. For antagonist studies, control responses were obtained to capsaicin (100 μmol/L in saline, aerosolized for 15 seconds), acrolein (10 mmol/L in saline, aerosolized for 60 seconds), and DEP-OE (1 μg/mL in saline, aerosolized for 60 seconds) before intravenous introduction of Janssen 130 (30 mg/kg, 1% methyl cellulose in saline) into the animal 60 minutes before repeat challenge with capsaicin, acrolein, and DEP-OE. At the end of the experiment, the conduction velocity of the single nerve fiber was measured to determine whether it was a slow-conducting nonmyelinated C-fiber or a fast-conducting myelinated Aδ-fiber. Using the same experimental setup with the vagus nerves left intact and in the absence of neuromuscular blockade, we assessed tracheal pressure (P_T_ Δ increase [cm H_2_O]) as a marker of airflow obstruction, which was expressed as the mean ± SEM.

### *In vitro* measurement of isolated vagus nerve depolarization

Guinea pigs and mice were killed by means of injection of sodium pentobarbitone (200 mg/kg administered intraperitoneally). Vagus nerves were dissected, and depolarization was assessed as a measure of sensory nerve activation, as described in previous publications.^24^, ^25^, ^26^ Human vagus was obtained from IIAM, as previously described (http://www.iiam.org/).

### Data analysis and statistics

Inhibition of DEP, phenanthrene, antimycin A, H_2_O_2_, capsaicin, and acrolein responses in the isolated vagus nerve preparation was analyzed by using a 2-tailed paired *t* test, comparing responses to agonist in the absence and presence of antagonist in the same piece of nerve. Data are presented as means ± SEMs, with statistical significance set at a *P* value of less than .05. In single-fiber experiments data were analyzed by using the paired *t* test, comparing responses (absolute values) after stimulus to baseline values immediately preceding the response. Data are presented as means ± SEMs, with statistical significance set at a *P* value of less than .05. Inhibition of fiber firing was analyzed by using a paired *t* test, comparing responses after antagonist with control values before antagonist or using an unpaired *t* test comparing responses with vehicle control responses, as appropriate.

## Results

### DEP-induced activation of airway sensory nerves

**Fig 1:**
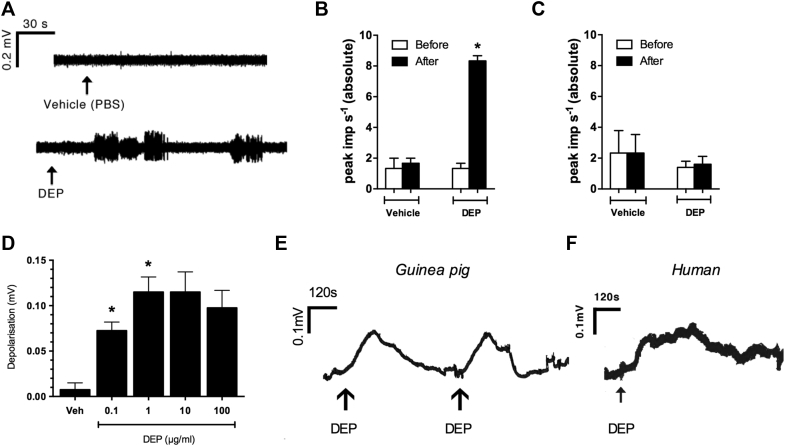
DEPs activate airway sensory afferents. **A,** Representative trace of action potential firing induced by vehicle (PBS) or DEPs (10 μg/mL administered intratracheally) recorded from a guinea pig and airway C-fiber afferent. **B** and **C,** Peak action potential impulses induced by vehicle (PBS) or DEPs in airway C-fiber (n = 3; Fig 1, *B*) and Aδ-fiber (n = 4; Fig 1, *C*) afferents. *P < .05, paired *t* test. **D,** DEP concentration response in isolated vagus nerve (n = 4). *P < .05, repeated-measures 1-way ANOVA with the Dunnett *post hoc* test compared against vehicle. **E,** Representative trace of depolarization induced by DEPs (1 μg/mL) in isolated guinea pig vagus nerve. **F,** Representative trace of depolarization induced by DEPs (1 μg/mL) in isolated human vagus nerve. Data in histograms are expressed as means ± SEMs.

**Fig E1:**
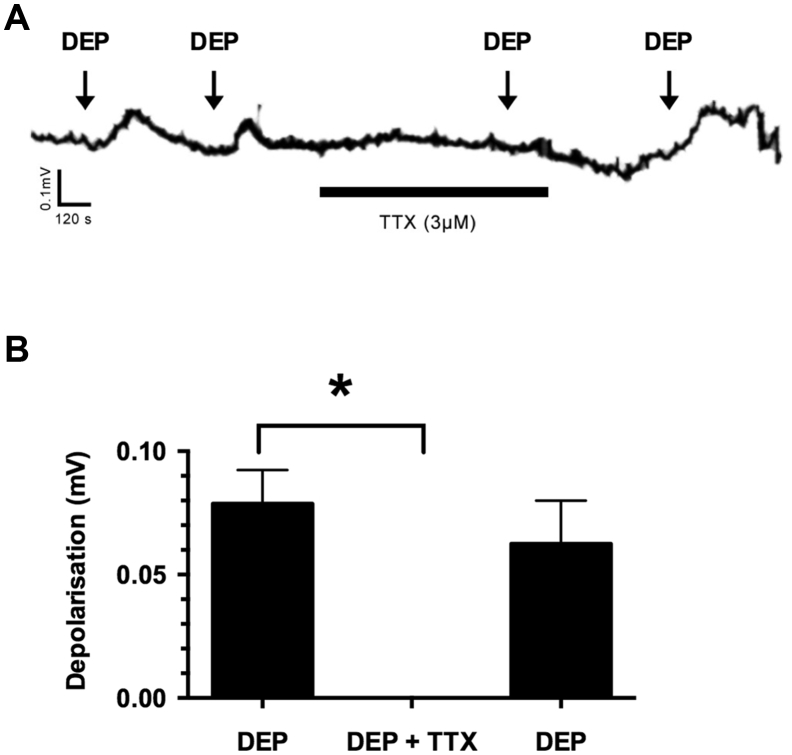
Effect of TTX on DEP-induced depolarization of guinea pig vagus nerve. **A,** Representative trace showing the effect of TTX (3 μmol/L) on DEP (1 μg/mL)–induced depolarization of guinea pig vagus nerve. Two control responses were obtained to DEPs (1 μg/mL) and DEPs in the presence of TTX, and recovery of the response to DEP after washout was determined. **B,** Summary bar graph showing initial depolarization to DEPs *(left column)*, followed by response obtained in the presence of TTX (3 μmol/L; all values, 0 mV) and a recovery response after washout (*right column*; n = 4). Data are expressed as means ± SEMs. *P < .05, paired *t* test.

Intratracheal instillation of DEPs (10 μg/mL in PBS; dose volume, 200 μL) in an anesthetized guinea pig model^23^, ^24^ evoked action potential firing in chemosensitive C-fibers (Fig 1, *A* and *B*) but not mechanosensitive Aδ-fibers (Fig 1, *C*, and Table I). The effect of DEPs was examined in an isolated vagal nerve preparation to investigate the mechanism further and provide translational data.^24^, ^25^, ^26^ DEP-evoked concentration-dependent depolarization of the guinea pig vagus (Fig 1, *D* and *E*), which was completely abolished in the presence of tetrodotoxin (TTX; which blocks the flow of sodium ions into nerve cells, a necessary step in the conduction of nerve impulses in excitable nerve fibers; 3 μmol/L; see Fig E1 in this article's Online Repository at www.jacionline.org). DEPs also depolarized isolated human vagal tissue in a similar manner to guinea pig tissue (Fig 1, *F* and *E*, respectively).

**Table I:** Effect of DEPs on airway-specific, afferent nerve fibers *in vivo* in the guinea pig

	C-fiber (CV ≤1 m/s [n = 3])	Aδ-fiber (CV >1 m/s [n = 4])	
DEP (10 μg/mL administered intratracheally)	7.33 ± 0.88*	1.6 ± 0.51	
Acrolein (10 mmol/L, aerosol)	8.67 ± 1.20*	1.6 ± 0.68	
Capsaicin (100 μmol/L, aerosol)	8.33 ± 0.33*	Capsaicin sensitive: 11.3 ± 4.05*	Capsaicin insensitive: 1.27 ± 0.37*

Peak action potential impulses (imp s^−1^) induced by DEPs (10 μg/mL administered intratracheally), acrolein (10 mmol/L in saline, aerosolized for 60 seconds), or capsaicin (100 μmol/L in saline, aerosolized for 15 seconds) recorded from airway C-fiber or Aδ-fiber afferents are shown. Data are presented as means ± SEMs, and analyzed by using a paired *t* test, comparing responses (absolute values) after stimulus with baseline values immediately preceding the response. *Asterisks* indicate statistical significance, which was set at a *P* value of less than .05.

### Characterization of DEPs

**Fig 2:**
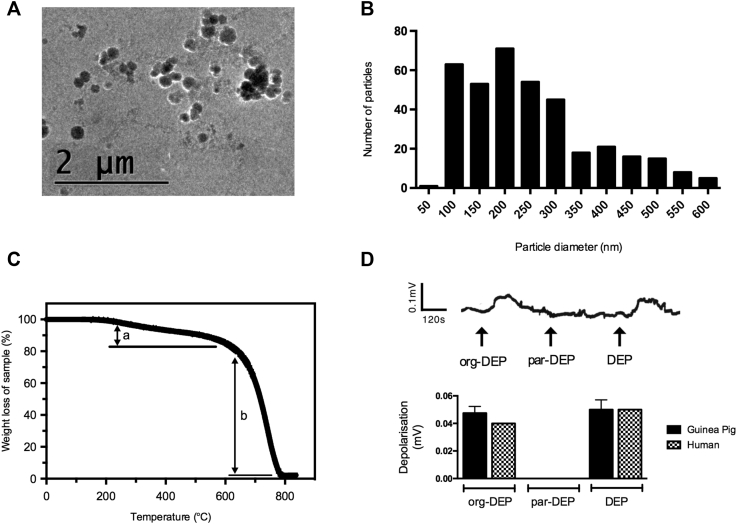
Physicochemical characterization of DEPs. **A,** Cryo-electron microscopy *(cryo-EM)* image of DEPs (1 μg/mL, Krebs-Henseleit solution). **B,** Size distribution of DEPs (1 μg/mL, Krebs-Henseleit solution), as measured by longest dimension, including agglomerates, derived from cryo-EM images (particle count, 394). Note that agglomerates larger than 600 nm were also present, although in low numbers (see Fig E2). **C,** TGA weight-loss profile of DEPs when heated to 850°C in air, showing the organic component fraction *(a)* and inorganic carbon fraction *(b)*. **D,** Example trace and summary data of the effects of org-DEPs (1 μg/mL), par-DEPs (1 μg/mL), and DEPs (1 μg/mL) in isolated guinea pig (n = 4) and human (n = 2) vagal tissue. Data are expressed as means ± SEMs for guinea pig. Depolarization of human vagal tissue was assessed in response to org-DEPs (0.03 and 0.05 mV) to par-DEPs (0 and 0 mV) and DEPs (0.06 and 0.04 mV).

**Fig E2:**
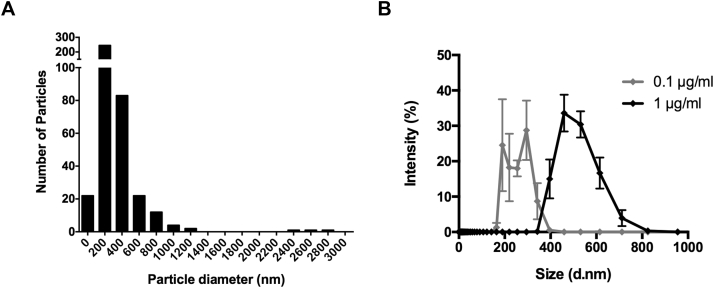
Size characterization of DEPs. **A,** Size distribution of DEPs (1 μg/mL, Krebs-Henseleit solution), as measured by longest dimension, including agglomerates, as analyzed by images produced by using cryo-electron microscopy *(cryo-EM)*. Particles were measured by longest length (n = 394). **B,** DLS hydrodynamic diameter measurements of DEP in Krebs-Henseleit solution (n = 3). Data are expressed as means ± SEMs.

**Fig E3:**
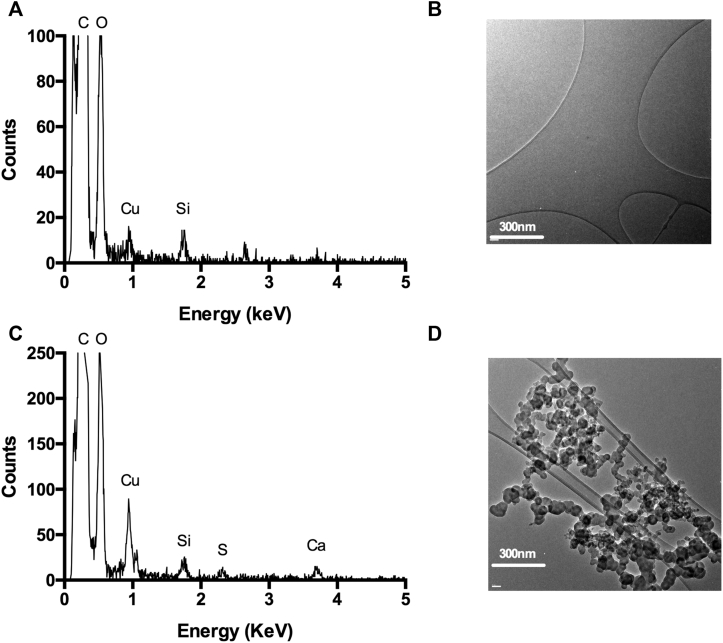
Metal species characterization of DEPs. **A** and **B,** TEM-EDX spectrum showing the element analysis profile of a blank carbon film area as a control (Fig E3, *A*), with the analyzed blank area of interest depicted (Fig E3, *B*). **C** and **D,** TEM-EDX spectrum showing the profile of detected elements in a DEP sample (Fig E3, *C*), with the analyzed sample of interest depicted (Fig E3, *D*). The trace of copper x-ray signals and part of the carbon signals are attributed to the holey carbon film–coated copper TEM grid and sample holder.

DEPs are made up of a carbonaceous core surrounded by an organic hydrocarbon component; however, the precise size and composition of each particle can vary greatly. Typically, DEPs consist of primary nanoparticles (<100 nm) that can form larger agglomerates several micrometers in size. The organic components of DEPs include polycyclic aromatic hydrocarbons (PAHs) and their derivatives, as well as traces of numerous transitional metals, including iron, vanadium, manganese, copper, zinc, and nickel.^27^ Cryo-electron microscopy images of the DEPs used in these studies (DEP SRM-2975, commercially available, generated by a forklift truck) indicated that individual primary nanoparticles were roughly spherical, with diameters of less than 100 nm; the majority were present as small irregular agglomerates, although larger agglomerates up to several micrometers were present (Fig 2, *A*). Particle size was quantified by measuring the longest length of the particles, and the majority were found to be less than 1 μm in size (mostly <600 nm; Fig 2, *B*, and see Fig E2, *A*, in this article's Online Repository at www.jacionline.org), which was confirmed by using DLS analysis (see Fig E2, *B*). Thus the DEPs were in a respirable format that would be expected to deposit throughout the lower respiratory tract on inhalation. TEM-EDX elemental analysis confirmed low levels of metals (see Fig E3 in this article's Online Repository at www.jacionline.org). TGA indicated that DEPs were composed of approximately 15% organic material and 83% inorganic carbon, with the remainder being trace impurities (Fig 2, *C*).

Given that DEPs appeared to activate chemosensitive rather than mechanosensitive sensory nerves, the organic chemical components of DEPs (org-DEPs) were separated from the cleaned particulate carbon core (par-DEPs) by using Soxhlet extraction. In both guinea pig and human vagal tissue, org-DEPs depolarized the vagus nerve in a similar manner to whole DEPs, whereas par-DEPs did not induce a response (Fig 2, *D*).

**Fig E4:**
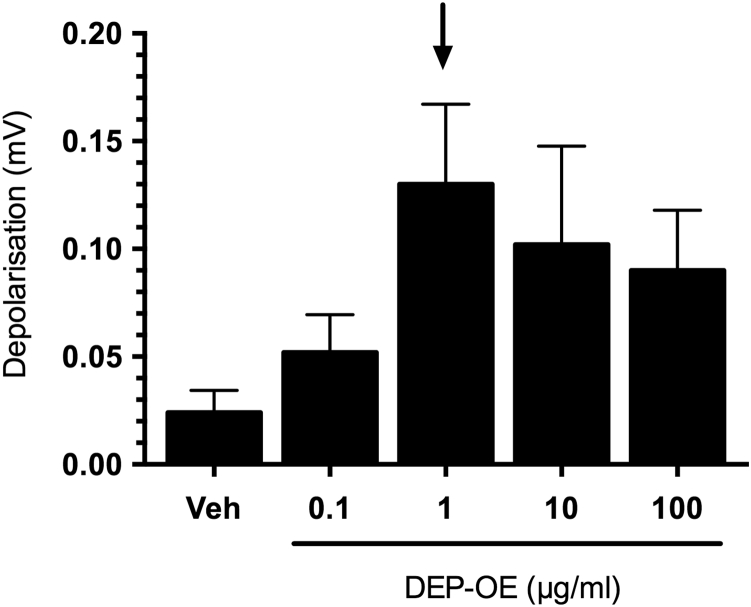
Effect of DEP-OE on isolated guinea pig vagus nerve. Concentration-dependent depolarization induced by DEP-OE in guinea pig isolated vagus nerve is shown. The *arrow* indicates concentration selected for subsequent antagonist studies (n = 4-5).

Having established that the organic extract of DEPs was responsible for its biological activity and to use a characterized and standardized supply, we used the commercially available extract of SRM-2975, namely SRM-1975, in the next experiments (this is referred to in this article as DEP-OE). DEP-OEs depolarized the guinea pig vagus nerve in a concentration-dependent manner similar to DEPs (see Fig E4 in this article's Online Repository at www.jacionline.org). These results indicate that the organic material embedded on the surfaces of DEPs contains the key components that activate sensory nerves. Polymyxin B (300 μg/mL), a cyclic cationic polypeptide antibiotic, is widely used to eliminate the effects of endotoxin contamination both *in vitro* and *in vivo* but had no effect on depolarization induced by DEP-OE (1 μg/mL; control, 0.1025 ± 0.009 mV; treatment, 0.1023 ± 0.020 mV; recovery, 0.0920 ± 0.014 mV).

### Role of TRP channels in DEP-OE–induced sensory nerve activation

**Fig 3:**
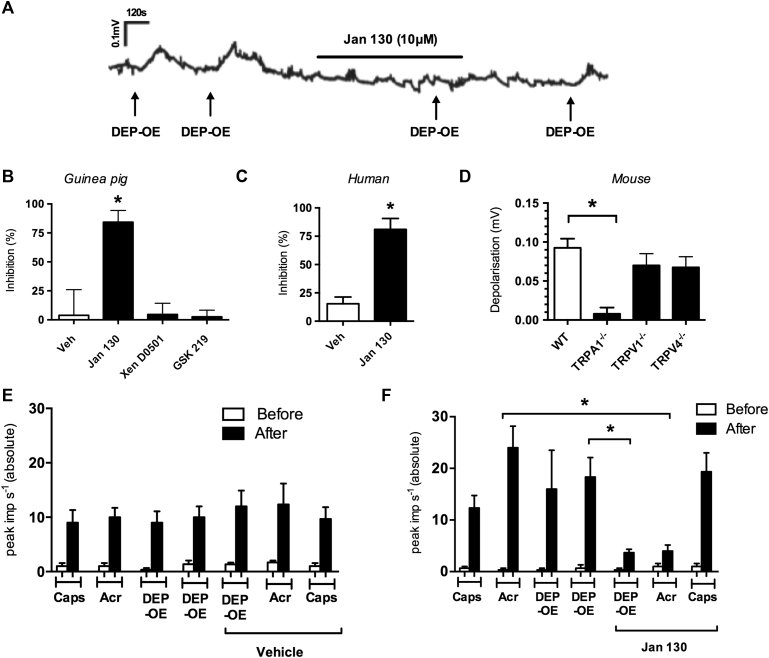
Effect of TRP antagonists on DEP-OE–induced vagal sensory nerve activation. **A,** Trace showing the effect of the TRPA1 antagonist (Janssen 130, 10 μmol/L) on DEP-OE (1 μg/mL)–induced depolarization of the guinea pig vagus nerve. **B,** Percentage inhibition of DEP-OE by vehicle (0.1% DMSO and antagonists: TRPA1, Janssen 130 [10 μmol/L]; TRPV1, Xention D0501 [100 nmol/L]; and TRPV4, GSK2193874 [10 μmol/L]) in guinea pig vagal tissue (n = 4-7). *P < .05, paired *t* test comparing antagonist responses with control responses in the same tissue. **C,** Percentage inhibition of DEP-OE–induced responses by Janssen 130 (10 μmol/L) in human vagus tissue (n = 3). *P < .05, paired *t* test comparing antagonist responses with control responses in the same tissue. **D,** DEP-OE depolarization in the isolated vagus nerve of TRP knockout mice (n = 4-6). *P < .05, unpaired *t* test. **E** and **F,** Effect of vehicle (0.5% methyl cellulose and 0.2% Tween in saline; Fig 3, *E*) or Janssen 130 (300 mg/kg administered intraperitoneally; Fig 3, *F*) on firing induced by DEP-OE (1 μg/mL, aerosol for 60 seconds), acrolein (10 mmol/L, aerosol for 60 seconds), and capsaicin (100 μ mol/L, aerosol for 15 seconds; n = 3) of guinea pig vagal C-fibers. *White bars* indicate peak impulses recorded immediately before application of agonists. *P < .05, paired *t* test. Data are expressed as means ± SEMs.

A submaximal concentration of DEP-OE (1 μg/mL) was selected for antagonist studies (see Fig E4). The specific TRPA1 antagonist Janssen 130 (10 μmol/L) significantly inhibited DEP-OE–induced depolarization in the isolated guinea pig vagus nerve (Fig 3, *A* and *B*), whereas vehicle (0.1% DMSO), the specific TRPV1 antagonist Xention D0501 (10 nmol/L), and the specific TRPV4 antagonist GSK2193874 (10 μmol/L) had no effect (Fig 3, *B*). Janssen 130, corresponding to the 130 compound of the patent WO2010/141805,^28^ also significantly inhibited DEP-OE–induced responses in the human vagus nerve (Fig 3, *C*). These results were confirmed by using genetically modified TRP knockout mice (Fig 3, *D*). Responses in TRPV1^−/−^ and TRPV4^−/−^ mice were not significantly different from those in wild-type (WT) mice. *In vivo* Janssen 130 (300 mg/kg administered intraperitoneally) significantly inhibited C-fiber firing to both the TRPA1-positive control (acrolein, 10 mmol/L) and aerosolized DEP-OE (1 μg/mL; Fig 3, *F*), whereas vehicle had no effect (Fig 3, *E*). Janssen 130 (300 mg/kg administered intraperitoneally) also significantly inhibited the increased tracheal pressure evoked by aerosolized DEP-OE (10 μg/mL; 2 responses to DEP-OE were evoked before [20.7 ± 3.02 and 19.63 ± 1.73 P_T_ Δ increase in cm H_2_O] and after [5.23 ± 0.79 P_T_ Δ increase in cm H_2_O] Janssen 130; n = 3; *P* < .05).

### Mechanisms of activation of TRPA1 by DEP-OE

**Fig 4:**
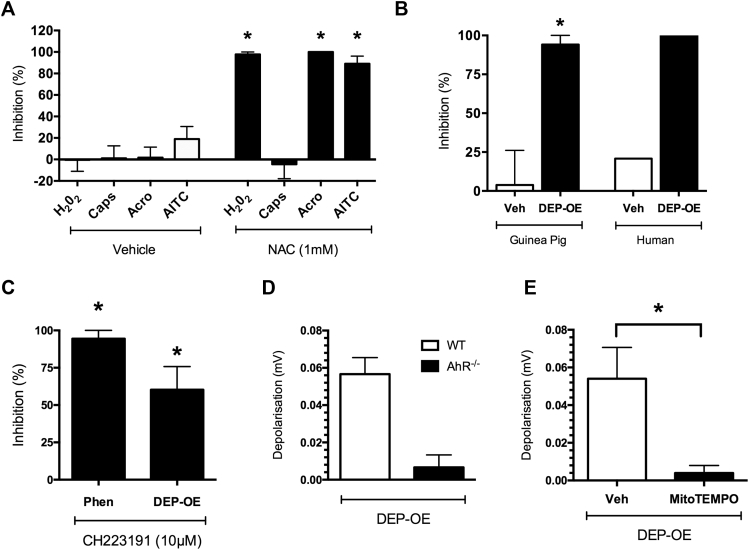
Mechanism involved in activation of TRPA1 by DEP-OE (1 μg/mL). **A,** Inhibition of H_2_O_2_ (10 mmol/L)–induced and TRPA1 agonist (300 μmol/L)–induced depolarization of guinea pig isolated vagus nerve by NAC (1 mmol/L; n = 4-7). *P < .05, paired *t* test comparing antagonist responses with control responses in the same tissue. **B,** Percentage inhibition of DEP-OE–induced depolarization by NAC (*black bars*, 1 mmol/L) in guinea pigs (n = 4). Depolarization to DEP-OE of human vagal tissue was assessed in the presence of vehicle (0.06 and 0.04 mV before compared with 0.05 and 0.03 mV after) or NAC (0.04 and 0.08 mV before compared with 0 and 0 mV after; n = 2). **C,** Percentage inhibition of phenanthrene (*Phen*; 1 nmol/L)– and DEP-OE–induced depolarization by the AhR antagonist CH223191 (10 μmol/L; n = 4-6) in guinea pig isolated vagal tissue. *P < .05, paired *t* test comparing antagonist responses with control responses in the same tissue. **D,** Depolarization induced by DEP-OE in isolated vagal tissue from AhR^−/−^ mice (n = 3). **E,** Inhibition of DEP-OE–induced depolarization by MitoTEMPO (2 μmol/L) in guinea pig isolated vagal tissue (m = 5). *P < .05, unpaired *t* test. Data are expressed as means ± SEMs.

**Fig E5:**
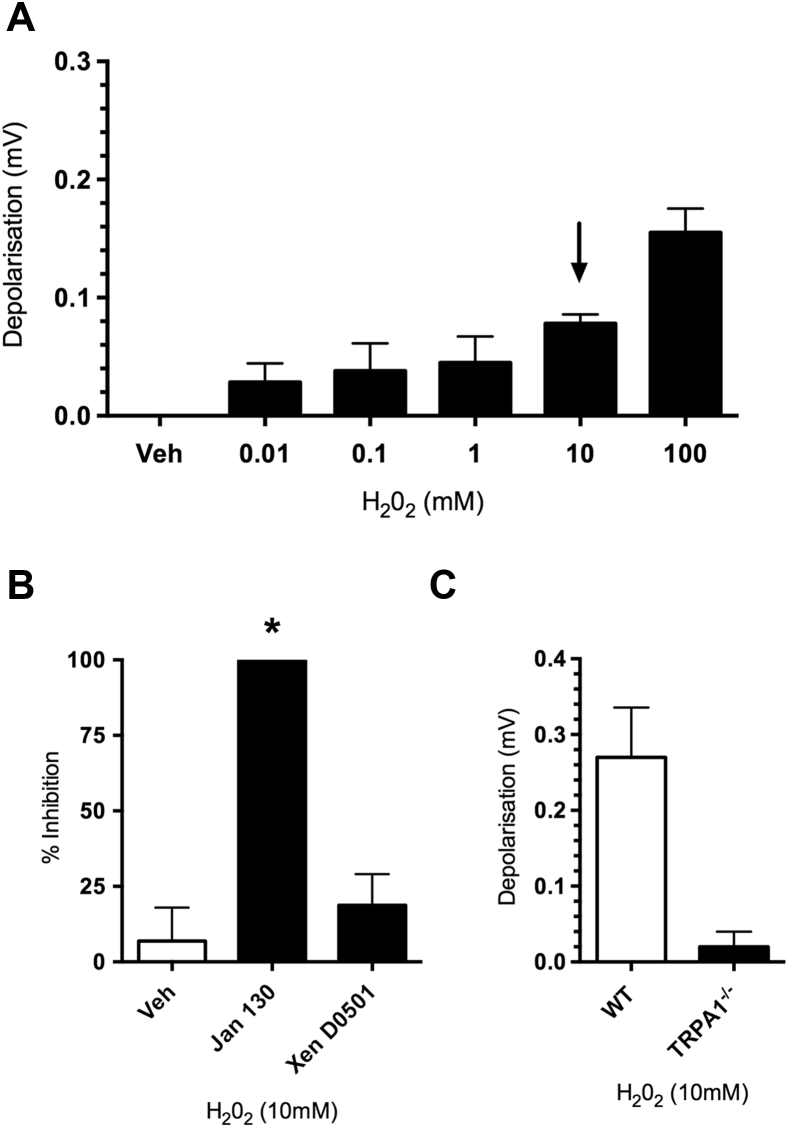
Depolarization induced by H_2_O_2_ on isolated vagus nerve and effect of pharmacologic interventions. **A,** Concentration response showing depolarization induced by H_2_O_2_ in guinea pig isolated vagus nerve. The *arrow* indicates the concentration selected for subsequent antagonist studies (n = 5-6). **B,** Effect of vehicle (0.1% DMSO), Janssen 130 (10 μmol/L; all values, 0 mV), and Xention D0501 (100 nmol/L) on H_2_O_2_-induced depolarization (n = 3-5). *P < .05, paired *t* test comparing antagonist responses with control responses in the same tissue. **C,** Effect of H_2_O_2_ on depolarization in vagal tissue from WT *(white bar)* and TRPA1^−/−^ mice (*black bar*; n = 2-3). Data are expressed as means ± SEMs.

**Fig E6:**
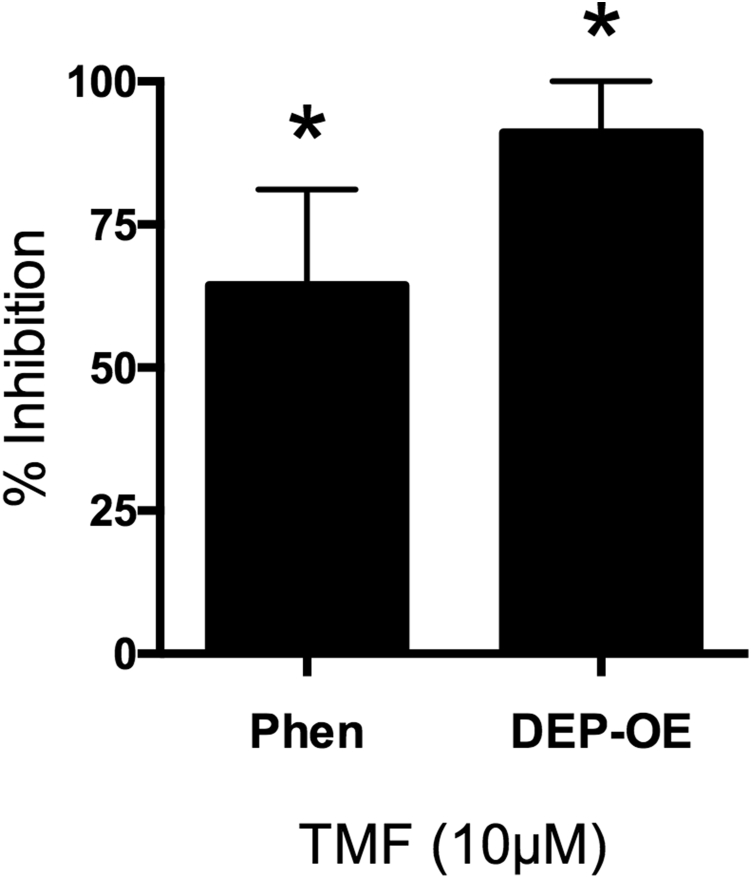
Effect of 2′, 4′-trimethoxyflavone *(TMF)* on phenanthrene *(Phen)*– and DEP-OE–induced depolarization of vagus nerve. Effect of TMF (10 μmol/L) on phenanthrene (1 nmol/L, n = 4)–induced and DEP-OE–induced depolarization (n = 6). *P < .05, paired *t* test comparing antagonist responses with control responses in the same tissue. Data are expressed as means ± SEMs.

**Fig E7:**
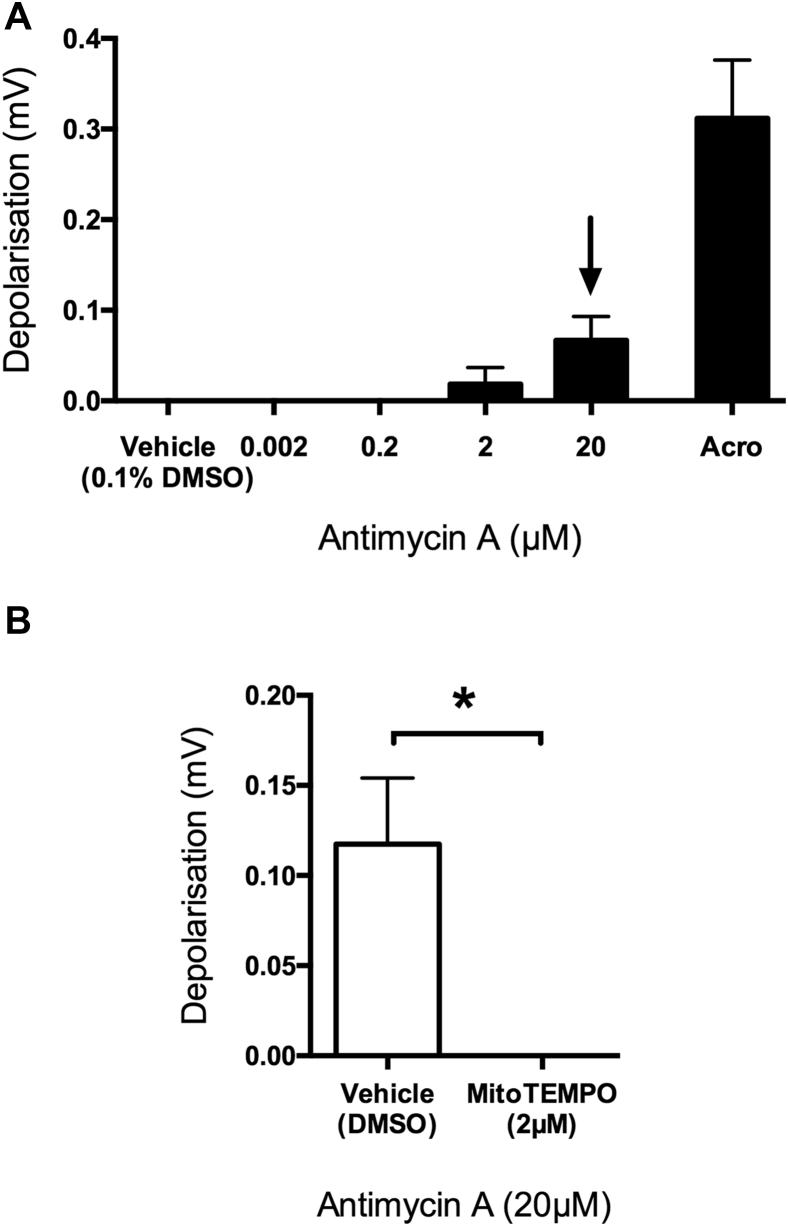
Effect of MitoTEMPO on antimycin A–induced depolarization of vagus nerve. **A,** Concentration response showing depolarization induced by antimycin A in guinea pig isolated vagus nerve. The *arrow* indicates concentration selected for subsequent antagonist studies (n = 5-6). **B,** Effect of vehicle (0.1% DMSO) and MitoTEMPO (2 μmol/L; all values, 0 mV) on antimycin A (20 μmol/L)–induced depolarization (n = 4). Data are expressed as means ± SEMs. *P < .05, unpaired 2-tailed *t* test.

The ability of DEPs to generate oxidative stress has been implicated as a key mechanism driving its adverse health effects.^29^ Oxidative stress and the production of electrophiles have been shown to activate TRPA1 through covalent modification of cysteine residues.^30^, ^31^, ^32^ The oxidant H_2_O_2_ depolarized isolated guinea pig vagus nerve in a concentration-dependent manner (see Fig E5, *A*, in this article's Online Repository at www.jacionline.org). Janssen 130 (10 μmol/L) but not vehicle (0.1% DMSO) or TRPV1 antagonism significantly inhibited H_2_O_2_-induced depolarization in the guinea pig vagus nerve (see Fig E5, *B*). Vagus nerves from TRPA1^−/−^ mice had significantly reduced depolarization responses to H_2_O_2_ compared with tissues from WT mice (see Fig E5, *C*). In the presence of the antioxidant N-acetyl cysteine (NAC; 1 mmol/L), responses to H_2_O_2_ (10 mmol/L), acrolein (300 μmol/L), and allyl isothiocyanate (AITC; 300 μmol/L; TRPA1 agonists) were abolished (Fig 4, *A*). DEP-OE–induced depolarization was also abolished by application of NAC on guinea pig and human vagus (Fig 4, *B*). When referencing the wide range of electrophilic chemicals present within DEP-OE, phenanthrene (a PAH) was identified to be present in relatively high concentrations (SRM-1975 Certificate Analysis Sheet, NIST). PAHs are traditionally thought to exert their toxic effects through induction of the aryl hydrocarbon receptor (AhR), a well-conserved transcription factor. Application of 2 specific AhR antagonists, CH223191 (10 μmol/L) and 2′, 4′-trimethoxyflavone (10 μmol/L), significantly inhibited depolarization responses to both phenanthrene (1 nmol/L) and DEP-OE (Fig 4, *C*, and see Fig E6 in this article's Online Repository at www.jacionline.org). Depolarization of the vagus nerve by DEP-OE was reduced in AhR^−/−^ mice compared with WT mice (Fig 4, *D*). Furthermore, antimycin A, a mitochondrial electron transport chain inhibitor and generator of mitochondrial oxidant stress, depolarized the vagus nerve in a concentration-dependent manner (see Fig E7, *A*, in this article's Online Repository at www.jacionline.org), and this response could be inhibited by the mitochondrial superoxide scavenger (2-[2,2,6,6-Tetramethylpiperidin-1-oxyl-4-ylamino]-2-oxoethyl)triphenylphosphonium chloride (MitoTEMPO; 2 μmol/L; see Fig E7, *B*). MitoTEMPO was capable of reducing the depolarization induced by DEP-OE (1 μg/mL) compared with that with vehicle controls (Fig 4, *E*). These data suggest that electrophilic compounds, such as the PAHs present in DEPs, activate TRPA1 through an oxidative stress mechanism that involves AhR and generation of mitochondrial oxidative stress.

### Translational experiments with generator diesel

**Fig 5:**
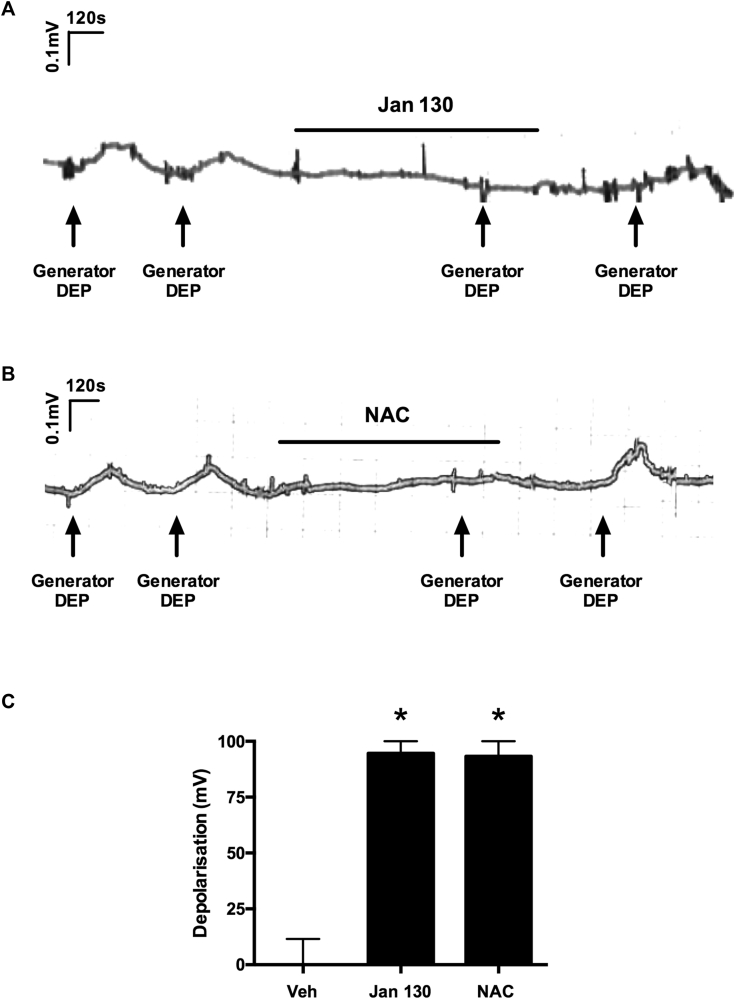
Effect of Janssen 130 and NAC on generator DEP–induced depolarization of guinea pig vagus nerve. **A** and **B,** Trace showing the effect of the TRPA1 antagonist Janssen 130 (10 μmol/L; Fig 5, *A*) or the antioxidant NAC (1 mmol/L; Fig 5, *B*) on generator DEP (1 μg/mL)–induced depolarization of the guinea pig vagus nerve. **C,** Summary graph of effect of vehicle (0.1% DMSO), Janssen 130 (10 μmol/L), and NAC (1 mmol/L) on generator DEP (1 μg/mL)–induced depolarization (n = 3-5). *P < .05, paired *t* test comparing antagonist responses with control responses in the same tissue. Data are expressed as means ± SEMs.

In the studies presented we used a characterized and standardized supply of commercially available DEP (DEP SRM-2975, generated by a forklift truck). However, we also repeated key observations with generator DEPs, which have been aged and diluted to mimic real-world occupational and environmental conditions. This generator diesel has been used in controlled human exposure studies and is associated with a range of respiratory symptoms.^10^, ^11^, ^22^ In these experiments depolarizations of the guinea pig vagus evoked by generator DEPs (1 μg/mL) were inhibited by the TRPA1 antagonist Janssen 130 (10 μmol/L; Fig 5, *A* and *C*) or the antioxidant NAC (1 mmol/L; Fig 5, *B* and *C*), confirming results obtained with DEP SRM.

## Discussion

Epidemiologic studies have found strong associations between exposure to DEPs and respiratory symptoms, including cough, wheeze, and shortness of breath.^6^, ^7^ In addition, links have been made between combustion-derived PM and asthma symptoms and exacerbations.^33^ DEP exposure has also been strongly associated with acute worsening of lung function^34^ and airway hyperreactivity in asthmatic subjects.^35^ Although a large number of previous studies have focused on the inflammatory effects of DEPs on airway epithelium and immune cells,^36^, ^37^ it is still not known how DEPs can evoke respiratory reflexes and the associated symptoms or by what mechanisms.

Initial studies confirmed our hypothesis that intratracheal instillation of DEPs could directly activate airway sensory afferent nerves. However, contrary to our expectations that PM would activate mechanosensitive rapidly adapting receptors (RARs) or Aδ-fibers, it was the chemosensitive C-fibers where action potential discharge was noted in response to DEPs. The isolated vagal preparation was used to confirm these observations and to investigate this mechanism further because it is an *in vitro* technique amenable to precise pharmacologic study without the complications often associated with the interpretation of *in vivo* experiments.^16^, ^24^, ^25^, ^26^, ^38^ Furthermore, and importantly, the use of the human vagus nerve preparation allowed generation of translational data and provided an early indicator that data generated in guinea pig vagus were predictive of effects in human subjects. The potential for DEPs to directly activate airway sensory nerves has important implications, given that millions of subjects living in urban environments are exposed to DEPs on a daily basis and that activation of airway sensory nerves can result in a wide range of respiratory symptoms that can be particularly debilitating for those with underlying respiratory conditions compared with healthy subjects.

Given its complex composition, DEPs (SRM-2975) underwent physicochemical characterization so that the biological data could be more easily interpreted. Cryo-electron microscopy imaging indicated that the majority of DEPs were present in small agglomerates, and TEM-EDX analysis indicated that a low level of metal impurity was present, which is in agreement with some previous studies.^39^ The TGA weight-loss profile revealed that the DEPs were composed of approximately 15% organic material, 83% inorganic carbon material, and 2% trace impurities, as measured by weight. These findings are in general agreement with existing published literature.^40^, ^41^ Soxhlet extraction of DEPs resulted in 2 separated components, the cleaned carbon particle core (par-DEPs) and the organic extract (org-DEPs). Only the org-DEPs activated the vagus nerve commensurate with an activity on the chemosensitive rather than mechanosensitive airway afferents. Other experiments have also highlighted the importance of the organic components of DEPs^40^, ^41^ in its bioreactivity *in vitro* and *in vivo*, although we acknowledge that the par-DEPs might be responsible for other biological effects of diesel.

TRP channels are environmental sensors and initiate activation of airway sensory nerves in response to exogenous and endogenous stimuli.^17^ Using pharmacologic intervention and tissues from genetically modified mice, we demonstrated that DEP-OE–induced activation of airway sensory nerves and increased tracheal pressure (indicative of airflow obstruction) was through TRPA1 activation. Although sensory afferents arising from the dorsal root ganglia are not airway innervating and calcium influx measurements do not assess action potential generation, our observations are consistent with data that demonstrated that DEPs could activate rodent dorsal root ganglia neurons.^20^ This key result was also demonstrated by using human vagus nerve. TRPA1 is expressed exclusively on airway C-fibers, is activated by a number of toxic environmental irritants, and has been shown to cause cough in both human subjects and guinea pigs.^25^ TRPA1 is also thought to be a key channel involved in the late asthmatic response in a rat model of allergic inflammation,^42^ and TRPA1 gene polymorphisms have been associated with childhood asthma.^43^ TRPA1 can be activated by electrophiles through covalent modification of cysteine residues on the cytoplasmic N-terminus of the channel,^44^, ^45^ and this explains its sensitivity to reactive species that have an innate oxidative potential or the ability to generate intracellular oxidative stress.^44^, ^46^ In the present study we confirm that H_2_O_2_ was also able to depolarize the vagus nerve in a TRPA1-dependent manner^30^, ^47^ and that responses to H_2_O_2_ and DEP-OE were inhibited by the general antioxidant NAC. This is in general agreement with previous studies in which the effects of DEPs have been inhibited by application of NAC.^11^, ^48^ DEPs, like other PM, are known to have a redox potential,^49^ and these results suggest that certain organic compounds with oxidative potential within DEP-OE activate TRPA1.

Identification of the specific compounds responsible for neuronal activation is important because it might allow for strategies to be developed to produce safer diesel emissions. Although the list of compounds present in DEP-OE in extensive certain classes of compounds appear to be likely candidates for the observed activation of TRPA1, PAHs are present on the surfaces of DEPs and are known to possess toxic and carcinogenic properties. Phenanthrene, one of the most commonly studied PAHs, is found in higher concentrations in DEP-OE compared with other chemicals. Phenanthrene depolarized the vagus nerve in a similar manner to DEP-OE, and this depolarization was blocked by a TRPA1 antagonist, suggesting this was one of the chemicals responsible for activation of TRPA1. However, given the number of chemicals within DEPs that share similar attributes, such as other PAHs or nitro-PAHs, it is unlikely to be the only activator of this pathway.

The toxic effects of PAHs are traditionally thought to be mediated by the cytosolic AhR, a highly conserved and expressed transcriptional regulator.^50^, ^51^ On ligand binding, AhR is transported to the nucleus whereby it heterodimerizes to the aryl hydrocarbon nuclear translocator and forms a complex so that the transcription of regulatory sequences that contain xenobiotic response elements can occur.^52^ Target genes include detoxification response enzymes, such as the widely studied CYP1A1 enzyme.^53^ After transcription has occurred, AhR is transported back to the nucleus and degraded. Previous studies in other experimental systems have shown that PAHs present within DEPs can activate AhR signaling cascades,^54^, ^55^, ^56^ and DEPs containing greater PAH content induce greater cytotoxic responses in a human bronchial epithelial cell line.^57^ However, typically, these transcriptional events occur over time courses that span several hours, which is at odds with the present study, in which we have shown that AhR inhibition (either in vagal tissue from AhR knockout mice or using small molecule inhibitors) immediately and significantly reduced depolarization that occurred in response to DEP-OE and phenanthrene. Because AhR inhibition had no effect on the TRPA1 agonist acrolein, it is likely that AhR plays a role upstream of TRPA1. Interestingly, AhR has been identified in the mitochondria, where it has been associated with mitochondrial reactive oxygen species (ROS) production, and these effects are thought to be independent of either CYP1A1 or CYP1A2.^58^ Vagal sensory fibers are densely packed with mitochondria.^48^ Antimycin A, which evokes ROS from mitochondrial complex III, has been demonstrated to evoke action potential discharge from nociceptive C-fiber terminals innervating the mouse airways in a TRPA1-dependent manner.^59^ In this study we confirmed that antimycin A evoked vagal sensory nerve activation and that this and the effect of DEP-OE were inhibited by the mitochondrial superoxide scavenger MitoTEMPO. For the first time, these unique findings describe a nontranscriptional signaling pathway for AhR and a role for mitochondrial ROS in activation of airway sensory nerves and initiation of respiratory reflex events evoked by urban air pollution.

To assess the physiologic relevance of the DEP SRM used experimentally with regard to real-life situations, we also repeated key observations with generator DEPs that have been aged and diluted to mimic real-world occupational and environmental conditions. In these experiments depolarization of the guinea pig vagus evoked by generator DEPs was inhibited by the TRPA1 antagonist or the antioxidant NAC, confirming results obtained with DEP SRM. This generator diesel has been used in controlled human exposure studies and is associated with a range of respiratory symptoms.^10^, ^11^

In conclusion, in this study we demonstrate a direct interaction between DEPs and airway C-fiber afferents mediated through an oxidative stress pathway and activation of the TRPA1 ion channel expressed on airway afferents. PAHs, major constituents of DEPs, are implicated in this process through activation of AhR and subsequent mitochondrial ROS production, which is known to activate TRPA1 on nociceptive C-fibers. These findings provide the first mechanistic insights into how exposure to a significant component of urban particulate air pollution might precipitate respiratory symptoms, such as cough and bronchospasm. A comparison between PAH content of diesel fuels and wider pollutants and TRPA1-mediated activation of airway sensory nerves will lead to further insights into the mechanisms driving the harmful effects of air pollution on the respiratory tract and mitigation strategies for those who are affected and at risk.

## Methods

Male Dunkin-Hartley guinea pigs (Harlan, Bicester, United Kingdom) were used, weighing 300 to 500 g for *in vitro* experiments and 400 to 750 g for *in vivo* single-fiber experiments. Male C57BL/6 mice (Harlan) weighing 18 to 20 g were used as a background to produce WT mice. TRPV1^−/−^ breeding pairs were backcrossed onto the C57BL/6 background and were obtained originally from Jackson Laboratories (Bar Harbor, Me). TRPA1^−/−^ mice were originally developed by Professor David Julius (UCSF) and supplied as backcrossed 10 generations onto the C57BL/6 background by Professor Peter Zygmunt from Lund University. Homozygous breeding pairs of mice genetically modified to disrupt the TRPV4 gene were obtained from Riken BioResource Center (Tsukuba, Japan).E1, E2 Vagal tissue was obtained from AhR^−/−^ mice provided as a generous gift by Dr Rebecca Dearman from the University of Manchester and Dr Mark Hayes (Imperial College, London). Genetic knockouts were confirmed by means of genotyping. All animals were housed in a climate-controlled room (21°C) with food and water freely available for at least 1 week before commencing experiments. All experiments were performed in accordance with the UK Home Office guidelines for animal welfare based on the Animals (Scientific Procedures) Act of 1986 and the Animal Research: Reporting of *In Vivo* Experiments guidelines.^E3^

### Compounds and materials

DEPs from a forklift truck (DEP, SRM-2975) and its commercial organic extract (DEP-OE, SRM-1975) were purchased from NIST. Capsaicin (TRPV1 agonist), acrolein (TRPA1 agonist), allyl isothiocyanate (AITC; TRPA1 agonist), citric acid, methylcellulose, PBS, hydrogen peroxide (30% vol/vol, H_2_O_2_), NAC, CH223191, antimycin A, MitoTEMPO, polymyxin B sulfate salt, dichloromethane (DCM), and DMSO were purchased from Sigma-Aldrich (Dorset, United Kingdom). 2′, 4′-Trimethoxyflavone was purchased from Tocris Bioscience (Bristol, United Kingdom). Janssen 130 (TRPA1 antagonist), corresponding to compound 130 of the patent WO2010/141805 A1, and GSK2193874 (GSK219, TRPV4 antagonist) were synthesized by the Medicinal Chemistry Department in Almirall (Almirall S.A., Barcelona, Spain). Phenanthrene was a generous gift from Dr Ian Mudway (Kings College London, London, United Kingdom). Xention D0501 (TRV1 antagonist) was provided by Ario Pharma (Cambridge, United Kingdom). Drugs were made up in stock solutions by using DMSO, with the final concentration of DMSO kept at 0.1% for experiments.

### Particle suspensions

Particle suspension solutions were freshly prepared daily. Suspensions of DEPs or par-DEPs were prepared in modified Krebs-Henseleit solution (NaCl, 118 mmol/L; KCl, 5.9 mmol/L; MgSO_4_, 1.2 mmol/L; NaH_2_PO_4_, 1.2 mmol/L; CaCl_2_, 2.5 mmol/L; glucose, 6.6 mmol/L; and NaHCO_3_, 25.5 mmol/L) by means of sonication in a water bath for 10 minutes before dilution to working concentrations. For *in vivo* experiments, suspensions were prepared in PBS in a similar manner.

### Physiochemical characterization of DEPs

#### Electron microscopy

The cryopreparation was done with a Leica GP automatic plunge freezer (Leica Microsystems, Milton Keynes, United Kingdom). Nanoparticles dispersed in 1 μg/mL in Krebs-Henseleit solution were dropped onto a grid and frozen by rapidly plunging into liquid ethane. These were transferred in their frozen state into a cryo-rod (Gatan 914, Gatan Inc, Pleasanton, Calif) and then into the electron microscope. Electron microscopy was carried out on a Jeol 2100F (JEOL USA Inc, Peabody, Mass) equipped with a Schottky Field Emission Gun and operated at 200 kV.

For chemical analysis, DEP samples were dispersed by means of sonication in ethanol and then pipetted onto a grid at room temperature. TEM-EDX analysis was performed on an FEI Titan 80-300 (S)TEM operated at 300 kV fitted with a Cs (image) corrector and SiLi EDX spectrometer (EDAX, Leicester, United Kingdom). EDX was carried out by condensing the electron beam onto the region of interest and acquiring spectra for 180 seconds.

#### TGA

The organic/inorganic ratio composition of SRM 2975 was assessed by using TGA with a PerkinElmer Pyris 1 machine (PerkinElmer, Beaconsfield, United Kingdom), heating 1.8 ± 0.2 mg of DEPs to 100°C under airflow (flow rate, 10 mL/min) and holding them isothermally for 30 minutes to remove residual water, solvent, or both. The temperature was then increased from 110°C to 850°C at a constant ramping rate of 10°C/min under air flow (10 mL/min).

#### DLS

DLS measurements were carried out by using a ZetaSizer Nano ZS (Malvern, United Kingdom). DEP samples were prepared in Krebs-Henseleit solution at 0.1 and 1 μg/mL and sonicated briefly in a sonicating water bath immediately before being placed into disposable microcuvettes. Samples were warmed to 37°C and left to equilibrate for 60 seconds before DLS measurements, which were carried out at a 173° backscatter angle and in triplicate for each sample. Measurement run durations were automatically calculated on a per-sample basis.

### Component separation DEPs

One gram of DEPs was loaded into an extraction thimble and extracted overnight with DCM by using Soxhlet extraction equipment at 40°C. The extracted solvent was then evaporated by using a rotary evaporator and reconstituted in DMSO up to a volume of 10 mL. This resulted in an “equivalent concentration” of organic extract of 0.1 g/mL, which represented the organic material present in 1 g of DEPs now being present in 10 mL of DMSO. The resulting org-DEP extract was centrifuged at 10,000*g* for 20 minutes to remove remaining particulates, and supernatant was carefully removed. DEP-OE was then diluted in DMSO and stored at −80°C until needed. The remaining particles left behind in the paper filter were heated gently overnight to completely evaporate any remaining DCM and were then kept as cleaned particles (par-DEP).

### *In vivo* recording of action potential firing in single-fiber afferents

Guinea pigs were anesthetized with urethane (1.5 g/kg) intraperitoneally. If required, anesthesia was supplemented with additional urethane. The trachea was cannulated with a short length of Portex tubing, and blood gases and pH were maintained at physiologic levels by using artificial ventilation (Ugo Basile small animal ventilator), with a tidal volume of 10 mL kg^−1^ and 50 to 60 breaths min^−1^ of laboratory air. The right jugular vein and carotid artery (passed to the ascending aorta/aortic arch) were cannulated for injecting drugs and measuring systemic arterial blood pressure, respectively. Systemic arterial blood pressure and heart rate were continuously recorded with a transducer (Gould P23XL, Gould Electronics, Eichstetten, Germany). Tracheal pressure was measured with an air pressure transducer (SenSym 647, Honeywell Inc, Minneapolis, Minn) connected to a side arm of the tracheal cannula. Body temperature was continuously monitored with a rectal thermometer and maintained at 37°C with a heated blanket and control unit (Harvard Apparatus, Holliston, Mass). Animals were paralyzed with vecuronium bromide initially administered at a dose of 0.10 mg/kg intravenously, followed every 20 minutes by 0.05 mg/kg administered intravenously to maintain paralysis. The depth of anesthesia was frequently assessed by monitoring the response of heart rate and blood pressure to noxious stimuli. Both cervical vagus nerves were located through a cervical incision and dissected free from the carotid artery and sympathetic and aortic nerves; both vagus nerves were cut at the central end. The left vagus nerve was used for sensory nerve fiber recording and was cleared of its surrounding fascia. The skin and muscle in the neck at either side of the incision were lifted and tied to a metal ring to form a well, which was filled with light mineral oil. Bipolar Teflon-coated platinum electrodes (exposed at the tips) were used for recording purposes by using fascia positioned on 1 electrode for a reference. The vagus nerve was placed on a small black Perspex plate to facilitate subsequent dissection. Thin nerve filaments were teased from the vagus nerve under a binocular microscope and placed on the second electrode until a single active unit or one of not more than 2 or 3 units was obtained. Action potentials were recorded in a conventional manner by using electrodes connected to a pre-amp headstage (Digitimer NL100K; Digitimer, Fort Lauderdale, Fla). The signal was amplified (×1000-×5000, Digitimer NL104), filtered (in the range of LF30Hz to HF8.5kHz, Digitimer NL125), and passed through a Humbug noise reducer (AutoMate Scientific, Albany, Calif) before input sampling and recording. All signals were sampled (50 kHz) and recorded by using the Spike 2 software data acquisition system through a CED Micro1401 interface (Cambridge Electronic Design Limited, Cambridge, United Kingdom). The software allowed pulse train counting over selected time periods. In addition, monitoring of the input signal to the Spike software was also carried out on a digital storage oscilloscope (Tektronix DPO 2012). The input signal was also fed through an audio amplifier to a loudspeaker.

Aerosols were generated by using an Aerogen nebulizer (Buxco Nebulizer Control-5; Data Sciences International [DSI], St Paul, Minn) connected to the ventilator and arranged so that inspired air passed through the medication chamber before entering the lungs of anesthetized animals through the tracheal cannula.

Single vagus nerve fibers were identified as originating from the major groups of airway sensory nerve endings, slowly adapting stretch receptors, RARs, irritant receptors, and Aδ fibers, which were further subdivided into those that were more acid and/or less capsaicin sensitive and with CVs lower than conventional RARs and pulmonary/bronchial C-fiber receptors by using several criteria.^E4^ These include a pattern of spontaneous discharge, response to hyperinflation and deflation, adaptation indices, response to capsaicin/citric acid administration, and conduction velocities. As a rule, a receptor that had no obvious pattern to the spontaneous activity (often very sparse) and did not respond to hyperinflation/hyperdeflation but did respond to capsaicin aerosol was pursued as a C-fiber. Alternatively, a receptor that had a spontaneous discharge with a definite rhythmic respiration pattern and adapted rapidly/variably to hyperinflation/deflation was pursued as an Aδ-fiber.

After identification of a suitable single-nerve fiber, control responses were obtained to capsaicin (100 μmol/L in saline, aerosolized for 15 seconds), acrolein (10 mmol/L in saline, aerosolized for 60 seconds), and citric acid (300 mmol/L, aerosolized for 60 seconds). The nerves under investigation were then challenged with either vehicle (PBS, 200 μL) or DEPs (10 μg/mL in PBS, 200 μL, intratracheal dose), and subsequent action potentials were recorded. For antagonist studies, control responses were obtained to capsaicin (100 μmol/L in saline, aerosolized for 15 seconds), acrolein (10 mmol/L in saline, aerosolized for 60 seconds), and DEP-OE (1 μg/mL in saline, aerosolized for 60 seconds) before introduction of Janssen 130 (30 mg/kg, 1% methyl cellulose in saline) into the animal through the intravenous route 60 minutes before challenging again with capsaicin, acrolein, and DEP-OE.

At the end of the experiment, the conduction velocity of the single nerve fiber was measured to determine whether it was a slow-conducting nonmyelinated C-fiber or a fast-conducting myelinated Aδ-fiber. This was achieved by stimulating the vagus nerve close to the thorax with a suprathreshold voltage (Grass stimulator, 0.5 ms, 1 Hz). The corresponding action potential was recorded in the single nerve fiber under observation, and the time between the initial stimuli and the resulting action potential was calculated by using the Spike 2 software and used with the measured distance between the 2 electrodes to calculate the conduction velocity. All animals were killed at the end of experiments with an overdose of pentobarbitone.

### *In vitro* measurement of isolated vagus nerve depolarization

Guinea pigs and mice were killed by means of injection of sodium pentobarbitone (200 mg/kg administered intraperitoneally) and human vagus obtained from IIAM. The vagus nerves were dissected, and experiments were conducted in a fully characterized isolated vagus nerve preparation, as described in previous publications.E5, E6 The 2 vagus nerve trunks were then placed in Krebs-Henseleit solution bubbled with 95% O_2_/5% CO_2_ to stay oxygenated. The vagal tissue was then cleaned of connective tissue before being cut into 15- to 20-mm segments. These segments were then mounted into a grease-gap dual recording chamber system whereby the nerve was threaded through a narrow cylindrical gap between a test chamber and a reference chamber. The ends of the nerve exposed in each chamber were then electrically and chemically isolated by means of application of petroleum jelly into the cylindrical gap through a side arm. The end of the nerve exposed in the test chamber was then constantly perfused at a rate of 2 mL/min with oxygenated Krebs-Henseleit solution heated to 37°C by using a custom-designed water jacket housing. Borosilicate glass electrodes mounted onto an Ag/AgCl pellet (World Precision Instruments, Sarasota, Fla) were filled with Krebs-Henseleit solution and placed into contact with the nerve in both the test and reference chambers. Changes in surface charges between the test and reference chambers were then recorded by using an extracellular potential differential amplifier (DAM 50 Bioamplifier, World Precision Instruments). Nerve depolarizations were filtered at 0.3 kHz, amplified ×50, and recorded onto a chart recorder (Lectromed Multi-Trace 2). During experiments, the perfusate through the test chamber of the vagus was maintained at 37°C at a rate of 2 mL/min and a change of drug containing solution was made through a manifold. Subsequent vagal depolarization were recorded in response to various stimuli applied for 2 minutes. For general antagonist studies, 2 repeatable baseline responses to agonists were first obtained before pretreatment with an antagonist for 10 minutes before the 2-minute application of the agonist in the presence of the antagonist. After a 10-minute washout, a recovery response to an agonist was then obtained to confirm nerve viability at the end of the experiment. If a recovery response could not be obtained, data were disregarded. For MitoTEMPO antagonist studies using antimycin A, an irreversible binding electron transport chain inhibitor, and other agonists, nerve viability was confirmed at the beginning of the experiment by using acrolein (300 μmol/L) before a single application of agonists with or without antagonist present. Responses were compared across different tissues from the same animal.
